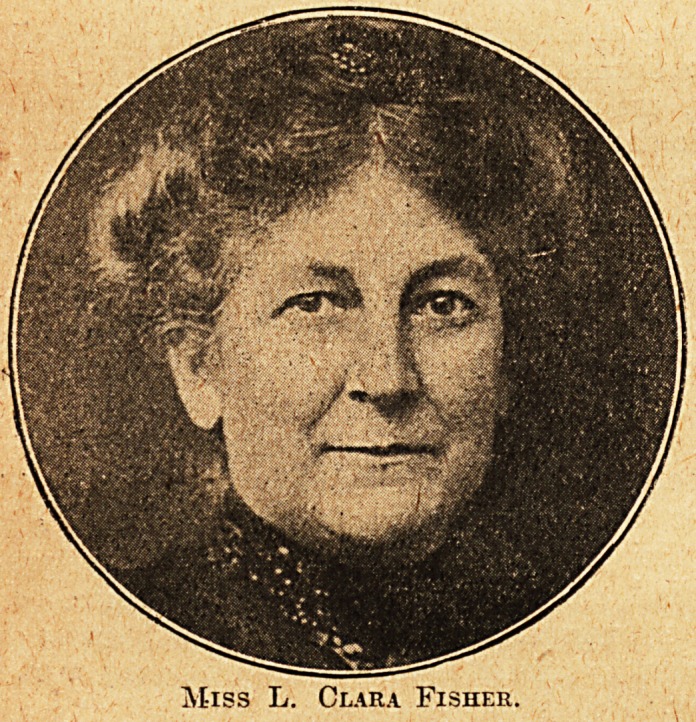# Round the Hospitals

**Published:** 1919-11-08

**Authors:** 


### ROUND THE HOSPITALS.

### 20,000 or 32,000 College Members: Which Shall It Be?

There is a very special reason why every intelligent present member of the College of Nursing should put her best foot forward and resolve to recruit one new member of the College by the middle or end of January 1920. If one-fourth of the present members of the College will decide to make a present of one new member to it by Christmas Day next, 20,000 fully-trained nurses might then enter on the New Year as members of the College. We are fully aware of the grave dissatisfaction which some matrons and sisters feel in regard to the special and continuous dry nursing of V.A.D.s of various types by some whose official object and duty it should be to put and keep the College of Nursing always first in all things. It is a matter of increasing discussion why a supreme worshipper of the V.A.D. should continue associated with the College. We make this bald statement because we think the time has arrived when the annoyance and irritation arising from this source, with increasing vigour lately, should be ended or mended forthwith. We feel ourselves in the position to express a hope and belief that if 32,000 is the number on the College register by the date of the next annual meeting, the troubles which have or may threaten the College will disappear. Then such vigour and energy may be put into the future conduct of its affairs as may astonish the nurses and excite the envy and wonder of their

ROUND THE HOSPITALS—(*continued*).

unenviable traducers. Wake up, nurses! Put your shoulders into the canvassing for new members of the College; stimulate all you know to help and co-operate, and 32,000 will be on the College register by the time of the next annual meeting in April. The action of the College in regard to the dishonouring salary the Birkenhead Guardians offered in their advertisement for a matron should demonstrate with great practical force, that all intelligent nurses can properly feel **The College**—and not a lawyer-exploited trades-union degenerator—**is the friend and ally of every British Nurse of Intelligence.**

Miss L. C. Fisher, whose portrait we publish, is a notable lady, one of those whose prudence and farsightedness contributed to build up that bulwark of protection for the nursing profession, the Royal National Pension Fund for Nurses. When this great organisation was first started many nurses felt doubtful about entrusting their savings to a new fund, even though founded expressly for their benefit. Miss L. Clara Fisher had no doubts. She was actually the first nurse to enter the newly-opened office doors and deposit her proposal. But,

Miss L. Clara Fisher.

as some of our readers may know, this honour of being No. 1 was accorded to Miss Katherine Dunn. We learn that when the first few proposals were brought up for consideration it was felt to be fairer to number them in alphabetical order, and for this reason Miss Fisher, though first on the scene, became possessor of Policy 2. She remains a most loyal and devoted friend to the R.N.P.F.N. and a constant attendant at their meetings. Indeed, she ascribes her present comfortable circumstances to the fact that it was started while she was a worker. The members of the First Thousand were true pioneers, and the many thousands who have followed in their thrifty footsteps have cause to be grateful for the good example they set their profession.

Norwich Cathedral was the scene of a very impressive ceremony on October 18 when the nurses and aids who had served under the Norfolk branch of the British Red Cross and the Order of St. John gathered to offer a service of praise and thanksgiving in the cathedral. A banner, the gift of Lady Leicester, was presented to the Dean to be preserved in the cathedral. Later on 3,000 doctors, nurses, and members of the voluntary aid detachments gathered in the Agricultural Hall, where Sir Arthur Stanley paid a fine tribute to their war work and sketched the future work of the twin societies, now firmly united.

An appeal has been issued from the British Hospital for Mothers and Babies at Woolwich, S.E. 18, under the Council for the Promotion of the Higher Training of Midwives, for funds to build a National Training-School for British Midwives. The hospital at Woolwich was intended to be the nucleus of such an institution, and the sum of £23,000 is in hand towards the undertaking, as well as a three-acre freehold building site. It is hoped to begin building next March, but the sum in hand is inadequate and donations are much needed to ensure its completion within as short a time as possible. British midwifery is heavily handicapped at the present time by a shortage of training facilities for the candidates desirous of taking the C.M.B. examination, and the project ought to commend wide support.

The Ladies Guild of St. Thomas's Hospital has held its first meeting for six years, the annual gathering having been dropped during the War. Adeline Duchess of Bedford presided and the Hon. Secretary, Mrs. W. H. Battle, made an earnest appeal for more ladies to devote themselves to this good work. To be personally connected with the magnificent work carried on at St. Thomas's and permitted to lend it aid is an honour all might covet to share and we have no doubt there will be a widespread response to Mrs. Battle's appeal.

It will be remembered that the memorial to King Edward VII, erected in Edinburgh, took the form, by Queen Alexandra's request, of a home in Edinburgh for retired nurses, where, with their own private room or rooms, they could enjoy also the pleasures of community life. In winter the private rooms are heated by radiators, but each lady provides for herself the coal or gas used in addition. The high cost of fuel has fallen heavily on all whose income is not elastic, and the matron and nurses of the Glasgow Royal Infirmary have had the happy inspiration to show their friendship by organising a sale to cover the cost of coal this winter. It proved very successful and a substantial sum has been handed over for this purpose to the Committee, who aim at running the establishment on a self-supporting basis.

The opening of the first hostel for Lying-in Mothers in this country, out of the generous gift presented by the American Red Cross Society, is an event which ought to draw the cords of friendship between the two nations closer. Bermondsey was

**ROUND THE HOSPITALS**—(*continued*).

selected for the home as one of the boroughs most in need of this kind of help, and Mrs. Davis, wife of the American Ambassador, performed the ceremony of opening the new hostel in the Grange Road, on October 29. A pretty touch was introduced when the matron, herself wearing the Mons ribbon, carried a two-weeks old baby, who was wearing his father's Mons medal, to present Mrs. Davis with a bunch of white heather. In time such lying-in homes will be considered indispensable in all crowded localities, and the American gift should help to stimulate other municipalities.

---

WITH the passing of the familiar name of the Incorporated Society of Trained Masseuses and its re-emergement under the title of the "Chartered Society of Massage and Medical Gymnastics," the Society will pass into a new stage of existence. A petition praying for the grant of a Royal Charter has been presented to His Majesty and all petitions for or against such grant are to be delivered at the Privy Council on or before December 1. The Charter will incorporate the Society of Trained Masseuses and the Institute of Massage and Remedial Gymnastics, having for its object a higher grade of training, education and professional status for persons engaged in the practice of massage; to promote a uniform curriculum and standard of qualification. Thus the position of the trained masseuse as a professional worker will be clearly defined and placed henceforth upon definite and solid basis. The advantages for the registered members will include the use of the term "Registered Member"; the wearing of the badge of the Chartered Society; attendance at conferences, lectures and demonstrations; becoming club members, with use of the libraries; application through the Society's Registry for work, and benefit through the Members' Fund.

---

AN inspiring sermon was preached by the Rev. Ronald Symes, Vicar of Kendal, at the special service in Carlisle Cathedral, when the doctors, nurses, and members of the St. John and Red Cross Detachments of the city and county gathered to give thanks. A great proportion of those present wore their uniforms and decorations, and the scene was most impressive. They assembled at the Fratry, and, having been marshalled in processional order by Canon Rawnsley and their County Director, they walked to the Cathedral, headed by the doctors and chaplains, the Queen's nurses, and the infirmary nurses. Mr. Symes urged the Red Cross workers to continue their voluntary public work. There was far too much paid public work at the present time, he declared, and far too few unpaid philanthropists.

---

MUCH is said of the unemployed nurses at present waiting for work. But meantime many quite good posts in the country are advertised in vain in the nursing papers. Salaries for district nurses with C.M.B. certificate, run from £100 to £120, with furnished cottage, allowance for uniform, and either bicycle or pony-trap provided. It is remarkable that these posts remain unfilled month after month. Some of the villages waiting for a nurse are situated in delightful surroundings with no vacant habitation to be had for love or money, except that for the nurse. The work is seldom very arduous and alternates with periods when there is very little to do. Pleasant society can be had at will. And the nurse with a friend or relative to share her cottage would find such a post really attractive, for she would arrange her own work and hold a unique position in her village.

---

THE Conference held at Cardiff, under the ægis of the Priory for Wales, to consider a scheme for bringing about co-operation and co-ordination of the nursing services throughout Wales and Monmouthshire, broke up without coming to agreement on the matters proposed. The scheme is a very big one. It aims at linking up the Voluntary Nursing Association, the County Councils, and municipal health services with a view to increasing the facilities for training in the Principality, attracting more candidates to the service of the sick, and generally getting more nurses to work in Wales. Many who attended the Conference displayed a fear lest the good work in this direction they had been helping to forward should be interfered with under the new centralisation scheme proposed. The promotors were not prepared to show in what manner the central organisation could obtain supplies, and, as Dr. Owen Morris observed, "there was an ominous silence about the financial aspect of the whole scheme." If the function of the advisory committee be limited to giving advice and making recommendations for the consideration of the different authorities, without the power of the purse to persuade, it certainly seems as though the Ministry of Health might undertake this duty. A further meeting will be held to consider the proposed scheme.

---

THE people of Chiswick are beginning to be very sore and impatient at the delay of the District Council in getting to work over the projected Maternity Home. The Red Cross Hospital Committee handed over to the Council the hospital in the Bolton Road established for wounded and disabled soldiers, for use as a Maternity Hospital together with all the furniture and fittings, valued at over £1,000. The gift was accepted with some demur by the Council, who doubtless foresaw that it would cost a good deal to equip the building properly and a good deal more to keep it going. The need for the Maternity Home is, however, a crying one in that neighbourhood, and the Council ought to be stimulated to immediate action. The Ministry of Health has shown itself generous towards all well-planned schemes for child and mother welfare, and we understand that the building in question is in every way well suited to the purposes of a Maternity Home. We trust the Council will at once appoint a strong working committee to put the matter through.

**ROUND THE HOSPITALS**—(*continued*).

It has been decided by the Committee of the Paddington Infirmary to admit V.A.D. probationers who have put in two consecutive years' service in military hospitals for a three-years' training, instead of four. The question of war bonus has been settled and nurses on the permanent staff of the Board employed at the Paddington Military Hospital are to be paid the war bonus calculated on the basis of their pre-war salaries.

At Bristol and Clifton the nursing staff of the District Nurses' Society and Nurses' Home are to have their salaries raised from £75 to a minimum of £90 a year, and the extra cost necesitates the raising of an additional £300 a year, with the alternative of closing the work altogether in certain districts. A meeting was held at Clifton on the 14th ult. at which Alderman Sheppard took the chair and advocated the necessity for placing the Society on a contributory basis. The Rev. J. S. Willimott of Leeds gave a most interesting account of the development of nursing in Leeds on a contributory basis, members paying 5s. a year or 1d. a week. The success depended largely on the zeal of the collectors, and it had been proved during the last eight years that it could be worked at a profit. It was decided to appoint a Committee and introduce at Bristol a scheme similar to that at Leeds. There is little doubt that the future of district nursing lies in the adoption of a self-supporting system combined with good salaries for the nurses.

The Scottish have a real talent for administration, as may be seen by the extent to which they come through to the top in every branch of the public service. The new scheme proposed for the nursing service in Forfarshire and introduced by Dr. Sinclair, medical officer of the county, has the merit of being original, comprehensive and workable. At present only one-eighth part of the district is covered by the Voluntary Nursing Associations. Dr. Sinclair proposes to combine all the different authorities in the various burghs, and institute a county nursing service with a central nurses' home from which nurses would be sent out for duty into any part of the county. They would take over school inspection and visiting, undertake general nursing and midwifery, and be responsible for child welfare and the care of the tuberculous. He proposed that nurses should not be stationed in rural districts for more than three months at a time and then be brought "back to civilisation." The scheme was assented to by the representatives of the four districts of the county who met in Forfar and we hope to hear more of it. One thing is certain: they will require a good training centre for nurses and midwives.

We understand that three members of the nursing staff at the Royal Hospital for Incurables, Putney, have just retired, their combined services totalling eighty-four years. This is a circumstance difficult to parallel at the present time, and speaks much for the harmonious administration of the institution.

The slander suit brought by Nurse Mary Ruth Maunders against Mr. Charles Ingleton, J.P., of Sheerness, is of peculiar interest. Nurse Maunders was not a trained nurse in the ordinary acceptation of the word, for she did not possess a certificate of three years' general hospital training. She was, however, a registered midwife. She was appointed superintendent nurse at Sheppey Union Workhouse, and continued in that office when that institution was taken over by the military, the Guardians having failed to secure a military nurse. Mr. Ingleton first took exception to Nurse Maunders on account of her attending an officer's wife in her confinement by consent of the medical officer. He also objected to her attitude and behaviour when before the Board on the occasion of a dispute with the workhouse master, whom she spoke of as a "worm" and "a liar." Accordingly when a proposal came before the Board to appoint her charge nurse at the Isolation Hospital, Mr. Ingleton, asked why he voted against her appointment, made use of the words on which the accusation of slander was based, "she is not a fit and proper person to be nurse there." He denied having any ill-feeling towards the nurse and certainly did not mean to imply that the plaintiff was immoral or badly conducted or unable to nurse properly. The jury found that the words complained of were defamatory, but that the defendant was not actuated by malice. Judgment with costs was given for the defendant.

The annual Sale of Work in aid of the District Nursing Fund for the sick-poor at York was held in Treasurer's House on October 23. Lady Maxee opened the sale on the first day and was supported by a numerous company. The matron, Miss Wainwright, assisted at the stall for clothing for the poor, and Miss Morgan, the late matron, who was unable to be present, sent a donation. Mr. Gerald Hughes, who presided, recalled the devoted services of the revered late Dean of York and Lady Emma Purey-Cust, the founders of the Nursing Fund.

The East Riding Red Cross and St. John County Committee held a meeting at Hull a short time back to discuss the draft scheme of the Central Joint V.A.D. Committee in London and criticised it somewhat severely. There are several administrative points on which they strongly disagree with the Central Committee and they have the unanimous support also of the York Branch of the B.R.C.S. The proposal that after a certain date all detachments shall wear the same uniform and also joint badges is objected to by the County Committee on the ground that so much sentiment exists in the matter of uniform that the best plan would be to allow detachments to continue wearing the uniform of St. John or the Red Cross as the case may be, and that this would avoid extra expense. In spite of the undisputed advantages of centralisation it can easily be overdone and is never very popular when supplanting a freer system.

## Figures and Tables

**Figure f1:**